# Using Kinect v2 to Control a Laser Visual Cue System to Improve the Mobility during Freezing of Gait in Parkinson's Disease

**DOI:** 10.1155/2019/3845462

**Published:** 2019-02-20

**Authors:** Amin Amini, Konstantinos Banitsas

**Affiliations:** College of Engineering Design and Physical Sciences, Department of Electronics and Computer Engineering, Brunel University London, London, UK

## Abstract

Different auditory and visual cues have been proven to be very effective in improving the mobility of people with Parkinson's (PwP). Nonetheless, many of the available methods require user intervention and so on to activate the cues. Moreover, once activated, these systems would provide cues continuously regardless of the patient's needs. This research proposes a new indoor method for casting dynamic/automatic visual cues for PwP based on their head direction and location in a room. The proposed system controls the behavior of a set of pan/tilt servo motors and laser pointers, based on the real-time skeletal information acquired from a Kinect v2 sensor. This produces an automatically adjusting set of laser lines that can always be in front of the patient as a guideline for where the next footstep would be placed. A user interface was also created that enables users to control and adjust the settings based on the preferences. The aim of this research was to provide PwP with an unobtrusive/automatic indoor system for improving their mobility during a Freezing of gait (FOG) incident. The results showed the possibility of employing such system, which does not rely on the subject's input nor does it introduce any additional complexities to operate.

## 1. Introduction

Freezing of gait (FOG) is one of the most disabling symptoms in Parkinson's disease (PD) that affects its sufferers by impacting their gait performance and locomotion. FOG is an episodic phenomenon that introduces irregularities in the initiation or continuation of a patient's locomotion and usually occurs in later stages of PD where patients' muscles cannot function normally and appear to be still when they are trying to walk [1–4]. This makes FOG one of the most intolerable symptoms that not only affects PD sufferers physically but also psychologically, as it makes them almost completely dependent on others for their basic and daily tasks. Consequently, the patient's quality of life decreases, and the healthcare and treatment expenditures increase, as does the cost of the injuries caused [1]. It has been estimated that about 50% of PwP experience FOG incidents [5]. Moreover, it has been proven that visual and auditory cues can have a positive impact on the subject's gait performance during a FOG incident [6–8]. Visual cues such as laser lines can act as a sensory guidance trick that provides an external trigger, which, in turn, can initiate movement [7].

There has been much research conducted towards implementing apparatus and systems that can provide visual and auditory cues for PwP. In work done by Zhao et al. [9], a wearable system based on modified shoes was developed in order to cast a laser-based visual cue in front of PwP. The system consisted of a 3D printed add-on that included a red laser line projector and pressure sensors that detect the stance phase of a gait cycle and turn the laser pointer on. The unit provided the option to adjust the distance between the laser light strip and the subject's foot for the optimal effectiveness, depending on the user's preferences. The research provided a simple, yet effective approach towards providing visual cues for PwP with locomotion issues. Nonetheless, like any other approaches, this too has some limitations, such as the constant need to carry the shoe add-on, the batteries needed for the device, charging the batteries, and remembering to switch them on.

In another attempt [10], researchers evaluated the effect of visual cues using two different methods, including a subject-mounted light device (SMLD) and taped step length markers. It was concluded that using laser projections based on SMLD have promising effects on the PwP's locomotion and gait performance. The method required patients to wear a SMLD that some patients might find inconvenient to have or even impractical in some situations. Moreover, SMLD systems have stability issues and steadiness difficulties due to the subjects' torso movements during a gait cycle. As expected, the visual cues must be constantly enabled during a gait cycle, regardless whether they are needed or not.

In [11], although the SMLD method was employed, researchers added the 10 seconds on-demand option to the “constantly on” visual cue casting. This system was more sophisticated, consisting of a backpack having a remotely controllable laptop that made the subjects' mobility even more troublesome.

In other attempts [12, 13], a different approach was implemented by using virtual cues projected on a pair of goggles that is only visible to the patient. In [14], the effect of real and virtual visual cueing was compared, and it was concluded that real transverse lines casted on the floor are more impactful than the virtual counterparts. Nonetheless, using virtual cueing spectacles (VCS) eliminates the shortcomings in other techniques such as limitations in mobility, steadiness, and symmetry. VCS have also the advantage of being used in an external environment when the patient is out and about.

Moreover, several research studies have been conducted using virtual reality (VR) to assess the possibility of VR integration for Parkinson's related studies [15–20]. Nonetheless, as the VR technology blocks patients' view and makes them unable to see their surroundings, the usage of this is limited to either rehabilitation by implementing exercise-based games, FOG provoking scenarios, or the assessment of patients' locomotion rather than real-time mobility improvement using cues.

Although they are effective to some extent, these attempts tend to restrict the user either by forcing them to carry backpacks or wear vests containing electronics, or making them rely on conventional approaches such as attaching laser pointers to a cane [21], or laser add-on for shoes.

The hypothesis of this study, on the other hand, is to propose a different technique: casting parallel laser lines as a dynamic and automatic visual cuing system for PwP based on Kinect v2 and a set of servo motors suitable for indoor environments. As Kinect has been proven to be a reliable data feed source for controlling servo motors [22, 23], the Kinect camera was chosen for real-time depth data feed for this study. This paper also examines the possibility of using the Kinect v2 sensor for such purposes in terms of accuracy and response time.

This research uses subject's 3D Cartesian location and head direction as an input for servo motors to cast visual cues accordingly. This eliminates the need of the user intervention or trigger, and at the same time, the need to carry or wear any special equipment. Despite this approach being limited to environments equipped with the proposed apparatus, it does not require any attachments or reliance on PwP themselves, something that can be beneficial in many scenarios. The system comprises a Microsoft Kinect v2, a set of pant/tilt servo motors alongside a microcontroller based on Arduino Uno and two laser line laser pointers. A two-line projection was chosen so that the second traversed laser line could be used to indicate a set area for which the next step has to land. The system was tested in different conditions, including a partially occluded scene by furniture to simulate a living room.

## 2. Methods

During the initial testing phase, 11 healthy subjects were invited, consisting of both males and females ranging from ages 24–31, with the age mean of 27 and SD of 2.34, a mean height of 174.45 cm (68.68 inch) and SD of 8.31 cm (3.27 inch) ranging from 163 to 187 cm (64.17 to 73.62 inch). They were asked to walk in predefined paths: 12 paths per subject, walking towards the camera and triggering a simulated FOG incident by imitating the symptom while having the Kinect camera positioned at a fixed location. The subjects' skeletal data were captured and analyzed by the Kinect camera in real-time. The software was written in C# using the Kinect for Windows SDK version 2.0.1410.19000. The room that was used for conducting the experiments consisted of different pieces of living room furniture to mimic a practical-use case of the device. This not only yields more realistic results but also tests the system in real-life scenarios where the subject is partially visible to the camera and not all the skeletal joints are being tracked. To test and compare the Kinect v2's accuracy in determining both vertical and horizontal angles according to the subject's foot distance to the Kinect camera and body orientation, eight Vicon T10 cameras (considered as the gold standard) were also used to capture the subject's movements and compare those with the movements determined by the Kinect. The Vicon cameras and the Kinect v2 captured each session simultaneously while the frame rate of the recorded data from the Vicon cameras was down-sampled to match the Kinect v2 at approximately 30 frames per second.

At a later stage and following an ethical approval, there was a recruitment of 15 PwP (with the collaboration of Parkinson's UK) to test the system and provide feedback. This research was published separately in [22]. The more in-depth analysis and information with regard to this focus group can also be checked via [24].

### 2.1. Kinect RGB-D Sensor

Microsoft Kinect v2 is a time-of-flight (TOF) camera that functions by emitting infrared (IR) lights on objects, and upon reflection of the lights back to the IR receiver, it constructs a 3D map of the environment where the *Z*-axis is calculated via the delay of receiving IR light [25]. Kinect v2 introduced many features and improvements compared to its predecessor such as 1080p and 424p resolution at approximately 30 frames per seconds for its RGB and depth/IR streams, respectively, as well as a wider field of view [26]. The ability to track 25 joints of six subjects simultaneously enables researchers to employ Kinect v2 as an unobtrusive human motion tracking device in different disciplines, including rehabilitation and biomedical engineering.

### 2.2. Angle Determination

The Kinect v2 was used to determine the subjects' location in a 3D environment and localize the subject's feet joints to calculate the correct horizontal and vertical angles for servo motors. To determine the subject's location, Kinect skeletal data were used for joints' 3D coordinate acquisition. A surface floor can be determined by using the vector equation of planes. This is necessary to automate the process of calculating the Kinect's height to the floor that is one of the parameters in determining vertical servo angle:

(1) $$\begin{array}{c} Ax+By+Cz+D=0, \end{array}$$

where *A*, *B*, and *C* are the components of a normal vector that is perpendicular to any vector in a given plane and *D* is the height of the Kinect from the levelled floor. *x*, *y*, and *z* are the coordinates of the given plane that locates the floor of the viewable area and are provided by the Kinect SDK. *Ax*, *By*, *Cz*, and *D* are also provided by the Kinect SDK once a flat floor is detected by the camera.

**Figure 1:**
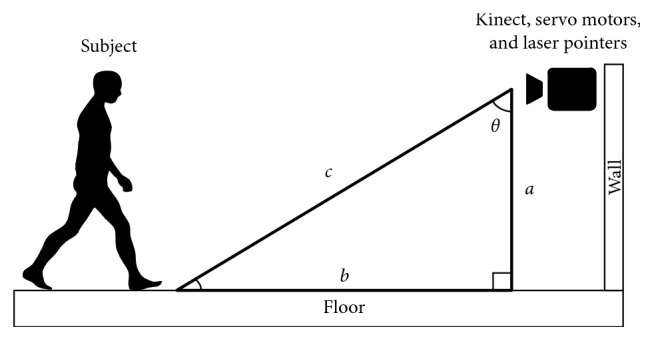
Vertical angle determination.

For vertical angle determination, a subject's 3D feet coordinates were determined, and depending on which foot was closer to the Kinect in the *Z*-axis, the system selects that foot for further calculations. Once the distance of the selected foot to the camera was calculated, the vertical angle for the servo motor is determined using the Pythagorean theorem, as depicted in Figure 1. The subject's skeletal joints' distance to the Kinect on the *Z*-axis is defined in a right-handed coordinate system, where the Kinect v2 is assumed to be at origin with a positive *Z*-axis value increasing in the direction of Kinect's point of view.

In Figure 1, *a* is the Kinect's camera height to the floor that is the same as variable *D* from equation (1) and *c* is the hypotenuse of the right triangle, which is the subject's selected foot distance to the Kinect camera in the *Z*-axis. *θ* is the calculated vertical angle for the servo motor. Note that we have considered the position offsets in the *X* and *Y* axes between the Kinect v2 camera and the laser pointers/servo motors in order to have the most accurate visual cue projection.

Our experiments showed that the Kinect v2 determines a joint's *Z*-axis distance to the camera by considering its *Y*-axis value; i.e., the higher the value of a joint's *Y*-axis is to the camera's optical center, the further the distance it has to the camera in the *Z*-axis. This indicates that unlike the Kinect's depth space, the Kinect skeletal coordinate system does not calculate *Z*-axis distance (Figure 1, variable *c*) in a perpendicular plane to the floor, and as a result, the height of the points, that in this case are joints, are also taken into consideration.

In case of a joint being obstructed by an object, for example, a piece of furniture, the obstructed joints' 3D Cartesian coordinate location tracking was compensated and predicted using “inferred” state enumerate, a built-in feature in the Kinect SDK. By implementing the “inferred” joint state, a joint data was calculated, and its location was estimated based on other tracked joints and its previously known location.

**Figure 2:**
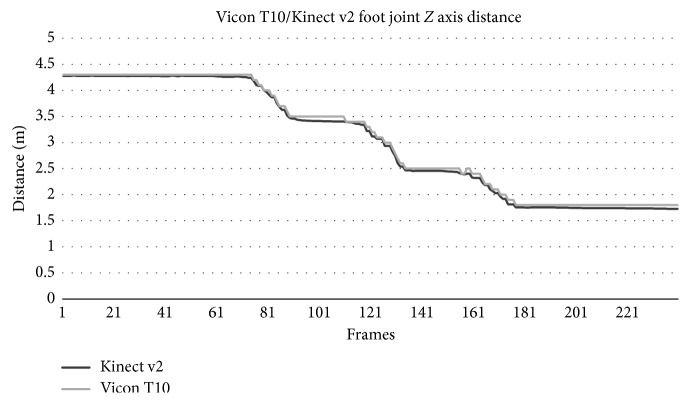
Subject's left foot distance to the camera in *Z-*axis using Kinect v2 and Vicon T10.

Figure 2 shows the Kinect v2 accuracy in determining a subject's joint (left foot) distance to the camera in *Z*-axis compared to a gold standard motion capture device (Vicon T10). It was concluded that Kinect v2 skeletal data acquisition accuracy was very close (98.09%) to the industry standard counterpart. The random noise artifacts in the signal were not statistically significant and did not affect the vertical angle determination.

The subject's body direction that determines the required angle for the horizontal servo motor can be yielded through the calculation of rotational changes of two subject's joints including left and right shoulders. The subject's left and right shoulder joints' coordinates were determined using skeletal data and then fed to an algorithm to determine the body orientation as follows:

(2) $$\begin{array}{c} \text{servo angle}=\left|90\pm \left(\sin^{-1}\left|\text{shoulder}A-\text{shoulder}B\right|\right)\right|. \end{array}$$

**Figure 3:**
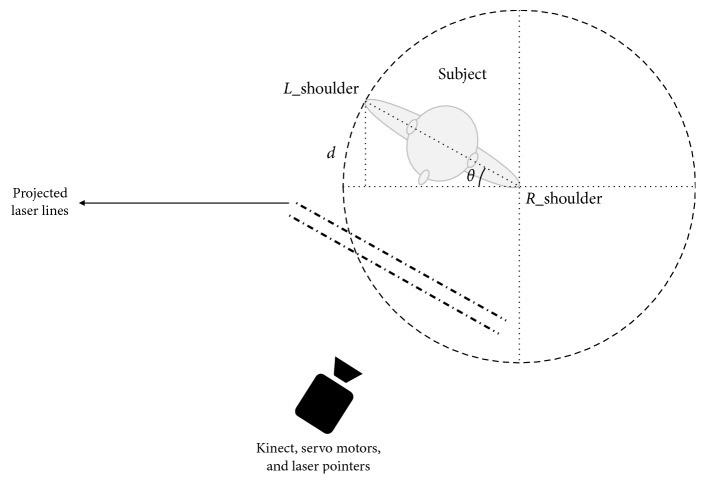
Horizontal angle determination (note that Kinect sees a mirrored image thus shoulders are reversed).

In Figure 3, *d* is the *Z*-axis distance difference to the camera between the subject's left and right shoulders.

Once *d* based in the equation (2) was calculated, the angle for the horizontal servo motor can be determined by calculating the inverse sine of *θ*. Depending on whether the subject is rotating to the left or right, the result would be subtracted or added from/to 90, respectively, as the horizontal servo motor should rotate in reverse in order to cast laser lines in front of the subject accordingly.

### 2.3. FOG Detection

**Figure 4:**
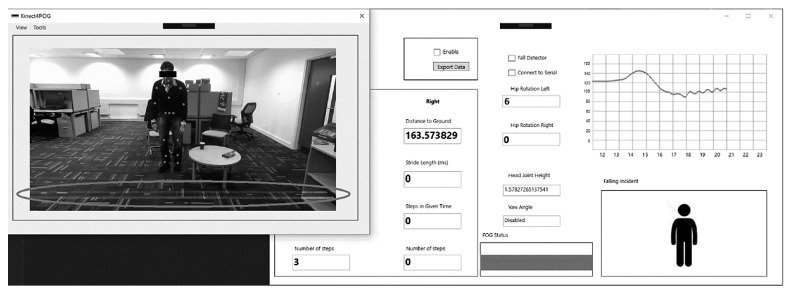
Graphical user interface for the developed software.

In previous studies, the authors have implemented the process of FOG detection in [27] using the gait cycle and walking pattern detection techniques [26, 28]. Once the developed system detects a FOG incident, it will turn the laser pointers on and start determining the appropriate angles for both vertical and horizontal servo motors. After passing a user-defined waiting threshold or disappearance of the FOG incident characteristics, the system returns to its monitoring phase by turning off the laser project and servo motors movements.  Figure 4 shows the GUI for the developed system application.

The left image shows a Parkinson's disease patient imitator during his FOG incident. The right window shows that the subject is being monitored, and his gait information is being displayed to healthcare providers and doctors. As it can be seen in the “FOG Status” section displayed in the bottom rectangle, the system has detected a FOG incident and activated the laser projection system to be used as a visual cue stimulus. The circled area shows the projection of laser lines in front of the subjects (according to the distance from their feet to the camera) and their body direction. The developed system also allows further customization, including visual cue distance adjustments in front of the patient.

### 2.4. Serial Connection

A serial connection was needed to communicate with the servo motors controlled by the Arduino Uno microcontroller. The transmitted signal by the developed application needed to be distinguished at the receiving point (the Arduino microcontroller), so each servo motor can act according to its intended angle and signal provided. We have developed a multipacket serial data transmission technique similar to [29]. The data was labeled at the transmitter side, so the microcontroller can distinguish and categorize the received packet and send appropriate signals to each servo motor. The system loops through this cycle of horizontal angle determination every 150 ms. This time delay was chosen as the horizontal servo motor does not need to be updated in real-time due to the fact that a subject is less likely to change his/her direction in very short intervals. This ensures less jittery and smoother movements of horizontal laser projection. The vertical servo motor movement was less prone to the jitters as the subject's feet are always visible to the camera as long as they are not obstructed by an object.

### 2.5. Design of the Prototype System

**Figure 5:**
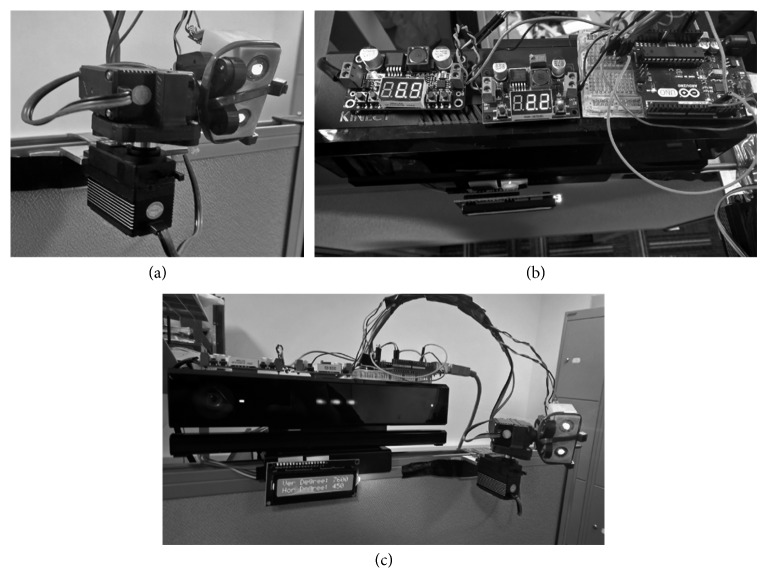
The developed prototype of the automatic visual cue system. (a) The two step motors controlling the horizontal and vertical alignment of the system. (b) A top view of the Kinect v2 combined with the microcontroller and voltage regulators. (c) A view of the prototype system in action.

A two-servo system was developed using an Arduino Uno microcontroller and two class-3B 10 mW 532 nm wavelength green line laser projectors as shown in Figure 5(a); green laser lines have been proven to be most visible amongst other laser colors used as visual cues [30]. A LCD display has also been added to the design that shows all the information with regard to vertical and horizontal angles to the user. Figure 5(a) shows the laser line projection system attached to the tilt/pan servo motors. Figure 5(b) shows the top view of the prototype system including the wiring and voltage regulators. Figure 5(c) shows the developed prototype system used in the experiment at different angles including the Kinect v2 sensor, pan/tilt servo motors, laser pointers, and the microcontroller.

## 3. Results

**Figure 6:**
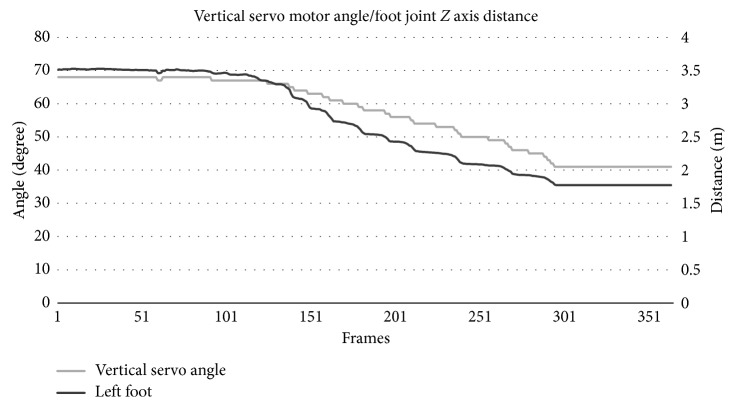
Vertical servo motor angle in relation to the subject's foot joint distance to the Kinect camera in *Z-*axis.

Figure 6 demonstrates the calculated vertical angle based on the subjects' feet/joint distance to the Kinect camera in *Z*-axis. The right foot has been omitted in the graph for simplicity.

As Figure 6 demonstrates, the system provided highly accurate responses based on the subject's foot distance to the camera in *Z*-axis and the vertical servo motor angle.

**Figure 7:**
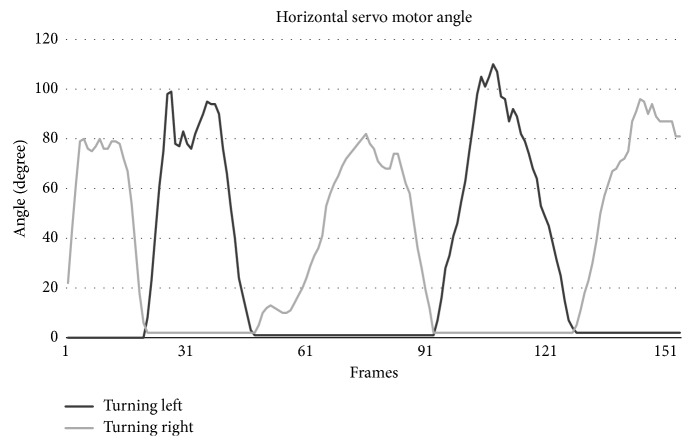
Horizontal servo motor angle changes according to the subject's body orientation and direction during a test.

Subjects were also asked to rotate their body in front of the Kinect camera to test the horizontal angle determination algorithm, and as a result, the horizontal servo motor functionality.  Figure 7 shows the result of the calculated horizontal angle using equation (2) for the left and right directions.

Figure 7 shows how the system reacts to the subject's body orientation. Each subject was asked to face the camera in a stand-still position while rotating their torso to the left and to the right in turns. As mentioned before, the horizontal angle determination proved to be more susceptible to noise compared to the vertical angle calculation. This is due to the fact that as the angle increases to more than 65 degrees, the shoulder farthest away would be obstructed by the nearer shoulder, and as a result, the Kinect should compensate by approximating the position of that joint. Nonetheless, this did not have any impact on the performance of the system.

Overall, the entire setup including the Kinect v2 sensor, tilt/pan servo motors, laser projectors, microcontroller, and LCD except the controlling PC will cost about *£*137.00, making it much more affordable than other less capable alternatives available on the market.

## 4. Discussion

A series of pan/tilt servo motors have been used alongside laser line projectors to create a visual cuing system, which can be used to improve the mobility of PwP. The use of the system eliminates the need to carry devices, helping patients to improve their mobility by providing visual cues. The implemented system has the ability to detect FOG using only the Kinect camera, i.e., fully unobtrusive, and provide dynamic and automatic visual cues projection based on the subject's location without the patient's intervention as opposed to other methods mentioned. It was observed that this system can provide an accurate estimation of the subject's location and direction in a room and cast visual cues in front of the subject accordingly. The Kinect's effective coverage distance was observed to be between 1.5 and 4 meters (59 and 157.48 inch) form the camera, which is within the range of the area of most living rooms, making it an ideal device for indoor rehabilitation and monitoring purposes. To evaluate the Kinect v2's accuracy in calculating the vertical and horizontal angles, a series of eight Vicon T10 cameras were also used as a golden standard. Overall, the system proved to be a viable solution for automatic and unobtrusive visual cues' apparatus. Nonetheless, there are some limitations to this approach including the indoor aspect of it and the fact that it requires the whole setup including the Kinect, servos, and laser projectors to be included in the most communed areas of a house such as the living room and the kitchen. Additionally, during the experimentation, the Kinect's simultaneous subject detection was limited to only one person. Nevertheless, Kinect v2 is capable to detect six simultaneous subjects in a scene. However, the laser projection system, in order to work properly, should only aim at one person at a time. The developed system has the ability to either lock on the first person that comes into the coverage area or distinguish the real patient based on the locomotion patterns and ignore other people. Despite that, the affordability and ease of installation of the system would still make it a desirable solution should more than one setup need to be placed in a house. Moreover, the use of a single Kinect would limit the system's visibility and visual cue projection as well.

## 5. Conclusion

The results of this research showed a possibility of implementing an automatic and unobtrusive FOG monitoring and mobility improvement system, while being reliable and accurate at the same time. The system's main advantages such as real-time patient's monitoring, improved locomotion and patient's mobility, and unobtrusive and dynamic visual cue projection make it, in overall, a desirable solution that can be further enhanced for future implementations.

As a next step, one could improve the system's coverage with a series of this implemented system to be installed in PwP's houses to cover most of the communal areas, or areas where a patient experiences the FOG the most (i.e., narrow corridors). One could also investigate the possibility of using such systems attached to a circular rail on a ceiling that can rotate and move according to the patient's location; this removes the need for extra setup in each room as the system can cover some additional areas. Moreover, by coupling the system with other available solutions such as laser-mounted canes or shoes, patients can use the implemented system when they are at home, while using other methods for outdoor purposes. This requires integration at different levels such as a smartphone application and visual cues in order for these systems to work as intended. Finally, the system's form factor can be made smaller to some extent by removing the Kinect's original casing and embedding all the equipment in a customized 3D printed enclosure, which makes it more suitable for a commercial production.

## Data Availability

The gait analysis data used to support the findings of this study are restricted by the Brunel University London Ethics Committee in order to protect patient privacy. Data are available from CEDPS-Research@brunel.ac.uk for researchers who meet the criteria for access to confidential data.
